# Women suffer but men die: survey data exploring whether this self-reported health paradox is real or an artefact of gender stereotypes

**DOI:** 10.1186/s12889-023-15011-4

**Published:** 2023-01-12

**Authors:** Susan P. Phillips, Madlen O’Connor, Afshin Vafaei

**Affiliations:** 1 Dept. of Family Medicine, Queen’s University, Kingston, ON Canada; 2 Dept. of Public Health Sciences, Queen’s University, Kingston, ON Canada

**Keywords:** Gender, Sex differences, Subjective health, Adults, Life expectancy

## Abstract

**Background:**

Despite consistently reporting poorer health, women universally outlive men. We examine whether gender differences in lived circumstances considered, and meaning attributed to SRH by women and men might explain this paradox.

**Methods:**

In an online survey 917 adults rated their health (SRH) and mental health (SRMH) and reflected upon what life experiences they considered in making their ratings. Descriptive findings were sex-disaggregated. The multiple experiences listed were then subject to factor analyses using principal components methods and orthogonal rotation.

**Results:**

Women reported poorer SRH and SRMH. They considered a wider range of circumstances, weighing all but self-confidence and behaviors as more important to SRH than did men. Two underlying components, psychosocial context and clinical status were identified overall. Physical health and pain were more important elements of men’s clinical status and behaviors. Comparisons with others of the same age played a larger role in male psycho-social context. Two components also underpinned SRMH. These were clinical problems and psycho-social circumstances for which self-confidence was only important among men.

**Conclusions:**

Women’s and men’s common interpretation of measures like SRH suggests that women’s health disadvantage is neither artefactual nor determined by gendered meanings of measures and does not explain the paradox. SRH and SRMH captured social circumstances for all. Convergence of characteristics women and men consider as central to health is evidence of the dynamism of gender with evolving social norms. The remaining divergence speaks to persisting traditional male stereotypes.

## Background

Women often describe poorer self-rated health (SRH) than do men, but when it comes to the most objective of all health indicators, they universally outlive men [1, 2]. To date, this incongruity remains unexplained. In particular, there has been minimal consideration of whether sex/gender differences in parameters contemplated when forming subjective ratings of health might give rise to poorer health ratings by women alongside better ratings by men. If, for example, men focus specifically on the presence of life-threatening illness while women additionally consider aspects of current well-being, then men’s subjective health ratings may more closely parallel life expectancy. The paradox of poorer female health ratings and greater longevity could then arise from how each group defines health. This matters for reasons that include, but go beyond explaining the paradox of women’s poorer SRH but greater longevity. A statistical assumption when analysing survey data is that the same construct is being measured in all participants and that those participants are independent of each other. This assumption necessitates examining whether personal judgements in rating health could be linked to social clusters or groupings. If sex/gender-based differences in the characteristics considered underlie apparent disparities in SRH that indicator will, in effect, be a different measure for women and men, which might partially explain the apparent SRH-longevity paradox.

We define sex as a set of biological attributes based on chromosomes, gene expression, hormones, and reproductive anatomy, and gender as the way life is shaped by socially constructed roles, behaviors, expressions and identities ascribed on the basis of sex [3]. We recognize that sex differences in the nature of chronic illnesses can contribute to differences in SRH. When we use the term gender we also acknowledge that sex and gender are entwined in how lives are lived, and in shaping and rating health [4].

Self-rated health has proven to be a reliable measure across gender, time and place [5, 6]. It appears to incorporate the impact of individual characteristics such as the presence of medical conditions and pain [7], health behaviors, physical function [8–10], and psychological well-being [11], along with sociodemographic influences like location, age, race/ethnicity, education, and income. The overall predictive value of SRH, however, is less reliable for women, older populations, and those of lower SES, a reality that is frequently noted but not explained [5, 12–18]. Although women's poorer SRH implies greater suffering, when morbidities are categorized men often report more life-threatening chronic diseases while women describe a greater number of disabling but not life-threatening conditions [14, 16]. Men’s focus on the presence of life-threatening diseases could make their SRH align more closely with life expectancy. Circumstances such as well-being, life satisfaction, mental health, or social connectedness might further differentiate gendered determinations of SRH. If, for example, collectively, men and women weigh social realities differently when rating health this could underpin the observed disparity in the SRH—longevity relationship [19]. Both biologic and social realities would then make SRH appear to be less predictive of female than male longevity. Women and men would have reliably described subjective health following divergent, group-based interpretations of the question, however aspects of health that are less directly related to mortality would have been more important constituents of SRH among women. The gendered variation in what each group considers when estimating SRH would produce heterogeneity in the meaning of this indicator. Its use as a predictor of life expectancy would be compromised because individual SRH would not be independent of the group effect of gender.

While subjective rating of health is a ubiquitous survey question, the dearth of attempts to clarify whether survey respondents attribute different meanings to the measure muddies interpretation of responses. Indirect assessments using cross-sectional data demonstrate, although not consistently, that mental health, physical function, pain, behaviors, and number and nature of chronic conditions do align with subjective health for women and men and do not explain observed sex differences in responses [4, 7, 8, 10, 20, 21]. US survey data examining associations of interactions between sex and other social or health circumstances [4] also concluded that men and women were remarkably similar in how they incorporated a range of chronic and acute health conditions, functioning, health-care utilization, and health behaviors in their SRH estimations.

To the best of our knowledge, only two studies have explicitly asked respondents to reflect upon the meaning of the measure, SRH, and what they would contemplate before making that rating. In 1994 Krause et al. [22] interviewed 158 adults and determined that characteristics considered by women and men when rating individual health were similar. In contrast, in 2012, Peersman [23] uncovered gender differences among 310 interviewees. Relative to women, men’s subjective health ratings focused more on physical function, negative health behaviors, and medical risks.

Years ago Kaplan et al. (1976) described SRH as a social construct [24] and more recently Jylha (2009) suggested that epidemiology has focused on statistical associations of variables rather than on the processes from which those variables emerge [8]. This speaks to the importance of maximising validity of measures and ensuring that, for our purposes, SRH represents a uniform and reliable construct for all participants. Idler and Benyamini (1997) reasoned that researchers are approaching the limits of what secondary analyses of large longitudinal data sets, often planned for other purposes, can tell us about the relationship between self-rated health and mortality [5]. They suggested that future research should include (1) outcomes other than mortality, (2) special populations, (3) more qualitative approaches and (4) the cognitive/cultural processes associated with assessments of health. Our aim was to address these ‘next steps’ (numbers 2–4) by examining the meanings of measures and, more specifically, whether ‘special population’ groups defined by sex/gender drive how and what individuals consider when locating themselves within the measure, SRH. Specifically, we will examine whether women’s and men’s considerations of individual and/or life circumstances differ when each group is asked to rate their health. There are two aspects to this: 1. measures that are interpreted differently by different groups (as explained above) and; 2. identifying missing correlates of SRH that have meaning for some or all groups – measures of, for example, reproductive and household work or control at home and work. These measures merit consideration because it may be that, for example, measures of control (at work or home) and, perhaps, of social capital, are more robust predictors of subjective health among one group than another. We address the first of these two aspects in this study and hope that others will address the second.

## Methods

### Design and study population

We conducted a survey, in English, of adults aged 25 + , gathering data from Sept. to Dec. 2021. Participants were recruited online via various networks (approximately 550 of all respondents) and via Amazon’s Mechanical Turks system. All responses were anonymous and could not be linked to specific individuals.

### Survey tool

The study was conducted using the Qualtrics platform (Qualtrics, Provo, UT). A consent statement was followed by a preamble stating: “Men consistently rate their own health better than do women, even though women consistently live longer than men. These ratings come from surveys that ask about self-rated health but rarely ask how participants chose that rating. In this questionnaire we are asking you the missing question—what do YOU think about when asked to rate your health?”.

### Measures

Demographic data collected included age, gender identity (male, female, other), and location by country (Canada, USA) or continent. Participants were asked to next rate their health (SRH) and mental health (SRMH) as excellent, very good, good, fair or poor. These measures were then dichotomized with excellent very good and good as the ‘good’ category and fair poor as the ‘poor’ category. Using broad constructs of physical function, mental health, disease diagnoses, lifestyle factors, and social/contextual circumstances identified by others as likely determinants of of SRH we then asked participants to rate the importance of each using a 5-point scale (not important = 0, to very important = 4). The circumstances, themselves, are listed in Table 2. Three subsequent questions asked which of a pair of characteristics was more important to SRH—current sense of health versus presence of chronic conditions, life-threatening versus chronic disabling conditions and finally, health relative to others of the same age versus absolute healthiness. A series of questions was asked specifically about SRMH (see Table 3).

### Analysis

Sex and age distributions in the total sample and by SRH and SRMH groups estimated the differences in distributions using Chi-square tests. Age was categorized as 25–44, 45–64 and 65 + . Although options offered for sex were male, female and other, the very small number of participants who described themselves as other precluded analysis of them as a subgroup. They are, nevertheless, included when characteristics of the whole study population are described.

The average ratings of each item describing its importance for inclusion in rating of SRH and SRMH were estimated and compared via student t-tests.

We performed factor analyses to identify the potential latent factors within the items that participants considered important in rating their overall and mental health. The principal components method was used to extract the potential underlying components, followed by an orthogonal rotation. The reason for choosing orthogonal solution over oblique solution was that the extracted components in data were not correlated. In initial analysis we followed Kaiser Criterion [25], which retains factors with eigenvalues greater than 1 and Cattell’s (1966) Scree test [26], which involves an examination of a plot of the eigenvalues for breaks or discontinuities, to determine the number of components to retain. However, because the 3-factor solution suggested by these methods was not interpretable and components had an eigenvalue marginally larger than 1 we continued the analysis with 2 components. In interpreting the component structure, an item was said to load onto a given component if the factor loading was greater than or equal to 0.50 and lower than 0.30 to the other component, also the difference between loadings onto the two components should be greater than 0.2. To establish a priori decision guidelines, components that did not meet the above criteria were excluded from analysis. The robustness of this exploratory factor analysis was assessed by estimating diagnostic measures of sampling adequacy (the Kaiser– Meyer–Olkin measure in which scores higher than 0.66 indicate adequate sample size for the conducted factor analysis) and by Bartlett’s test of sphericity (a hypothesis testing procedure to determine whether correlations between the variables, examined simultaneously do not differ significantly from zero, in other words to evaluate if the two retained components together are sufficient to explain correlations between items). All analyses used SPSS, version 27.

### Ethics and data access

The study received ethics approval from the Queen’s University Health Sciences Research Ethics Board (#6034322). All methods were performed in accordance with relevant guidelines and regulations and in accordance with the Declaration of Helsinki. The study purpose was explained in writing prior to participants moving on to the questionnaire. An explicit statement said that continuing to the questions implied informed consent to participate and for data analysis and publication. Participants were also told they could opt out completely at any time and that they could choose to not answer specific questions. The datasets generated and/or analysed during the current study are not publicly available as ethics approval allowed access only for the named researchers (the authors) however ethics amendments to make data available will be considered upon reasonable request made to the corresponding author. The study was supported via FutureGen – a Gender-Net Plus EU funded study in collaboration with the Canadian Institutes of Health Research (CIHR).

## Results

A total of 917 participants (male = 311, female = 596, other = 10) from Canada (43%), the US (41%), Europe (9%) and elsewhere (7%) responded to the questionnaire. Women’s SRH and, to a lesser extent, SRMH were significantly lower than men’s. Overall, older participants also reported poorer SRH but that relationship was not linear. Those aged 65 + rated their health as somewhat better than did those age 45–64. SRMH showed a consistent improvement with increasing age (Table 1).

**Table 1:** Sex and age distribution of participants and SRH, SRMH

		**Self-rated Health**		**Self-rated Mental Health**	
	Total	Good	Poor	Good	Poor
**Sex**					
**Women**	596 (44.6%)	505 (84.9%)	90 (15.1%)*	493 (83.0%)	101 (17.0%)*
**Men**	311 (35.1%)	282 (90.7%)	29 (9.3%)	268 (86.2%)	43 (13.8%)
**Other**	10 (1.1%)	6 (60%)	4 (40%)	4 (40%)	6 (60%)
**Age**					
**25–45**	409 (44.6%)	364 (89.0%)	45 (11.0%)	308 (75.5%)	100 (24.5%)*
**46–65**	322 (35.1%)	271 (84.4%)	50 (15.6%)	281 (87.5%)	40 (12.5%)
**65 + **	186 (20.3%)	158 (84.9%)	28 (15.1%)	176 (94.6%)	10 (5.4%)

^*^Chi-squared were significant at α level of 0.05

### Characteristics considered when determining SRH

Table 2 details the weightings participants gave to each listed characteristic in determining their SRH and SRMH. Women included a wider range of characteristics, overall, and generally considered each characteristic to be of greater importance than did men. Of the 14 options offered, the relative weightings of eight differed significantly between men and women. Six were weighed more by women while only self-confidence was significantly more important in men’s decision-making.

**Table 2:** Average ratings of importance of characteristics when considering SRH & SRMH (range 0 to 4)

Characteristics considered in rating health (SRH)					
Characteristic	Average overall	Average (women)	Average (men)	Average (25-45y)	Average (45 + y)
Physical ability^*†^	3.5	3.6	3.4	3.4	3.6
Pain^*†^	3.2	3.3	3.1	3.1	3.3
Mental health^*†^	3.1	3.2	2.9	2.9	3.2
Self-confidence^*‡†^	2.5	2.4	2.6	2.3	2.6
Current illness^*†^	3.2	3.3	3.0	3.1	3.3
Disabling conditions^*†^	3.3	3.4	3.1	3.1	3.4
Life-threatening conditions^*†^	3.3	3.4	3.1	3.2	3.4
Inherited health risks	2.5	2.5	2.5	2.4	2.5
Behavior -exercise, sleep, diet ^*†^	3.1	3.2	3.0	3.0	3.2
Substance use -smoking, drugs, alcohol	2.4	2.3	2.4	2.4	2.3
Life satisfaction^†^	2.7	2.7	2.6	2.5	2.8
Relationship with family^†^	2.4	2.4	2.4	2.2	2.6
Belonging to a community^†^	2.1	2.1	2.1	2.0	2.2
Age comparison	2.6	2.7	2.6	2.5	2.7
Characteristics considered in rating mental health (SRMH)					
Item	Average overall	Average (women)	Average (men)	Average (25-45y)	Average (45 + y)
Family^*†^	2.9	3.0	2.7	2.7	3.1
Friendship^*†^	2.8	2.9	2.7	2.7	2.9
Belonging	2.3	2.3	2.2	2.3	2.3
Physical^†^	2.9	2.9	2.8	2.7	3.0
Behaviors^*^	3.0	3.0	2.8	2.9	3.0
Substance Use^‡†^	2.0	1.9	2.1	2.2	1.9
Income	2.6	2.6	2.5	2.5	2.6
Psychiatric dx^†^	2.2	2.2	2.2	2.5	1.9
Confidence	2.7	2.7	2.7	2.8	2.6
Satisfaction	3.2	3.1	3.1	3.1	3.2

^*^t-test significant at the level of α = 0.05 between men and women

^†^t-test significant at the level of α = 0.05 between 25–45 and 45 +

^‡^the average was larger in men

The comparative importance of paired characteristics was relatively similar for both groups (men and women) and particularly so for ‘how healthy I think I am’ versus ‘health relative to others of the same age’ (*p* = 0.76). Men were somewhat, although not significantly more likely to consider life-threatening rather than disabling conditions (*p* = 0.18) in rating their health, but also to focus on how they felt at the moment instead of on having a life-threatening condition (*p* = 0.08).

To determine whether the 14 specific characteristics listed in the questionnaire were linked we next performed factor analyses (Table 3).

**Table 3:** Factor loading (overall, women, and men), Self-Rated Health

	All Participants		Women		Men		25–45		45 +	
	Component 1 Psycho-social	Component 2 Clinical	Component 1 Psycho-social	Component 2 Clinical	Component 1 Psycho-social	Component 2 Clinical	Component 1 (Psycho-social)	Component 2 (Physical)	Component 1 (Psycho-social)	Component 2 (Physical)
Physical	.158	.590	.142	.533	.209	.627	.130	.621	.161	.541
Pain	.091	.678	.095	.609	.090	.777	.076	.676	.079	.682
Mental	.579	.295	.563	.273	.640	.283	.527	.300	.609	.287
Confidence	.798	.080	.792	.093	.813	.099	.829	.037	.764	.127
Current_Illness	.056	.724	.021	.721	.098	.722	.011	.722	.061	.724
Disability	-.012	.795	-.025	.783	-.006	.818	-.018	.806	-.042	.783
Life_Threatening	.135	.734	.163	.681	.079	.811	.027	.803	.215	.653
Inherited	.507	.395	.497	.414	.505	.406	.491	.429	.521	.378
Behaviors	.507	.364	.481	.326	.582	.366	.452	.373	.538	.358
Substance Use	.352	.373	.338	.415	.365	.359	.344	.446	.371	.340
Satisfaction	.821	.001	.833	.027	.806	-.021	.815	.001	.822	-.008
Family	.875	-.028	.885	-.019	.852	-.046	.874	-.024	.875	-.038
Belonging	.838	-.018	.846	-.027	.812	-.008	.844	-.033	.828	.007
Comparison	.317	.147	.241	.126	.441	.169	.356	.077	.272	.218
Percentage of variance explained	28%	22%	27%	20%	30%	24%	28%	24%	28%	21%
Eigenvalue	4.6	2.3	4.5	2.2	5.0	2.5	4.4	2.7	4.6	2.2
KMO measure for sampling adequacy	0.87		0.85		0.86		0.85		0.85	
*P*-value of Barlett’s test of sphericity	< 0.0001		< 0.0001		< 0.001		< 0.0001		< 0.0001	

Two components emerged for the cohort as a whole. Considerations of mental well-being, self-confidence, life satisfaction, relationship with family, and sense of belonging together formed one component, which we will refer to as psycho-social context. Physical health, pain, current illnesses and disabilities, and life-threatening conditions mapped to a second component, referred to from here on as clinical status. Despite variations in loadings onto the two components no clear pattern was observed for the remaining items of inherited risk, behaviors, substance abuse, and age comparison, and none met a priori criteria to be included in a component. Therefore we concluded that these characteristics did not represent a component.

In general, factor analyses did not change when participants were stratified by sex or age. However, some differences were observed. Among men, physical health and pain contributed somewhat more to clinical status (Table 3). Men also weighed two items in the psycho-social component more than did women: behaviors and comparison with others of their age. Subtle variations with age were observed as well. For those aged 45 + , behaviors were a more important consideration when subjectively rating health (Table 3). Although comparison with others of one’s age did not meet a priori criteria, among those aged 25 to 45 it was more central to the psychosocial component (0.356 versus 0.272) than was so for older respondents.

### Self-rated mental health

Relationships with family or friends, and behaviors were significantly more important in women’s determinations of their SRMH (Table 2). The factor pattern for men and women was, again, generally similar. Overall, two components emerged from factor analysis of characteristics considered when rating mental health (Table 4).

**Table 4:** Factor loading (overall, women, and men), Self-Rated Mental Health

	All Participants			Women		Men		25–45		45 +	
	Component 1 Social Circumstances		Component 2 Clinical	Component 1 Social Circumstances	Component 2 Clinical	Component 1 Social Circumstances	Component 2 Clinical	Component 1 Social Circumstances	Component 2 Clinical	Component 1 Social Circumstances	Component 2 Clinical
Family	.796	-.079		.778	-.106	.812	.035	.815	.082	.771	-.046
Friendship	.830	-.050		.836	-.067	.812	.013	.827	.152	.828	-.092
Belonging	.723	.049		.742	.014	.667	.174	.703	.149	.761	.000
Physical	.638	.118		.648	.169	.580	.148	.485	.455	.616	.193
Behaviors	.618	.294		.605	.348	.592	.323	.427	.612	.622	.324
Substance Use	.261	.709		.270	.714	.186	.788	.137	.807	.240	.711
Income	.630	.258		.614	.245	.680	.280	.643	.334	.543	.356
Psychiatric dx	-.100	.829		-.180	.799	.061	.806	-.019	.614	-.161	.787
Confidence	.554	.395		.506	.435	.703	.216	.668	.145	.531	.447
Satisfaction	.602	.199		.623	.243	.612	.021	.675	.058	.590	.242
Percentage of variance explained	38%	16%		38%	17%	38%	16%	36%	18%	37%	17%
Eigenvalues	4.1	1.3		4.0	1.4	4.2	1.2	4.2	1.2	3.9	1.4
KMO measure for sampling adequacy	0.83			0.80		0.86		0.84		0.80	
*P*-value of Barlett’s test of sphericity	< 0.0001			< 0.001		< 0.001		< 0.001		< 0.001	

One was clinical problems such as substance abuse or having a psychiatric diagnosis. The other was psycho-social circumstances and included family relationships, friendships, sense of belonging, income, life satisfaction and, for men only, self-confidence (Table 4). Confidence was also important to the psycho-social circumstances domain among younger participants, while physical health was not. For this younger group, only, behaviors were part of the clinical problems domain.

Given that the Kaiser–Meyer–Olkin (KMO) measure of sampling adequacy for all two-component solutions was greater than 0.60 the size of the sample was deemed adequate. Furthermore, the statistical significance of Bartlett’s test suggested that the two-common factor solution was sufficient to explain the correlations.

## Discussion

Consistent with the repeated and sustained evidence cited above we found women’s health ratings to be poorer than men’s. Our main aim was to determine whether the paradox of women’s poorer subjective health but greater longevity might be artefactual, arising from women’s and men’s different interpretations when asked to subjectively rate their health. Specifically we examined whether gender differences in weighting of individual and contextual characteristics might shape women’s reports of poorer health, that is, whether men and women were, in effect, answering different questions when rating their health. Such a hypothesis is in keeping with others’ suggestions that men’s ratings focus on the presence or absence of life-threatening illnesses while women incorporate broader social and contextual circumstances that shape well-being or disability [14, 18, 27]. Spiers et al. (2003) also demonstrated that for older adults with disabling but no life-threatening conditions SRH remained a stronger predictor of mortality among men but that the relationship between SRH and mortality among all who reported life-threatening conditions was weak [16]. It may be that SRH is measuring something not captured by such individual-level circumstances as number and nature of chronic conditions, physical function or risky behavior. Findings from two small studies that directly examined what women and men consider when rating their health are contradictory. One demonstrated no sex differences in ‘frames of reference’ [22] while the other [23] found that men were more likely to consider negative health behaviors, physical health, physical function, medical risk factors and feeling good. The latter study suggests that gender-based differences in meanings attributed to subjective health do exist, but that their nature would not explain the paradox [23].

Participants appeared to consider similar characteristics when rating SRH and SRMH, although did so to different degrees. Women were more reflective overall, and thought more broadly and deeply about each possible determinant of individual health. Perhaps this deeper reflection moved women beyond the prevailing medical paradigm of equating poor health with the presence of disease. Alternatively, participants’ different ratings of health arising from consideration of the same circumstances may say that women’s lived realities are less positive than are men’s. Finally, male participants may have been less introspective and self-aware, defaulting to gender stereotypes of stoicism, denial of adversity and avoidance of delving into areas that might reveal weakness. For both SRH and SRMH possible explanations for women’s poorer ratings include the following: 1. that by asking only about characteristics already thought to shape SRH we have overlooked key constructs such as aspects of power and control at home and at work, or life-course effects of adversity in childhood; 2. that, as explored above, women are more reflective, in general, and when asked to rate their health they circumvent more immediate and more medical accounting of diseases and delve deeper; 3. that women’s perceptions or realities in some or all of the areas listed are more dire and predispose to poorer subjective health; and/or; 4. that relative to women, men are less introspective and self-aware, and default to the gender stereotypes of ‘stiff upper lip’, stoicism, denial of adversity, and avoidance of delving into areas that might reveal weakness [28].

Our findings do not invalidate SRH and SRMH as indicators of health but do highlight a few statistically significant gender differences in how each group framed meanings of those measures. The only characteristics that men weighed more than did women were self-confidence and, to a lesser and not significant extent, substance use. For women, and in contrast to some previous cross-sectional findings [14, 16, 18, 27], physical ability, pain, mental health, current illness, disabling conditions (i.e. current clinical status), but also life-threatening illness, and behaviors shaped their health rating significantly more than was so for men. In factor analysis equivalent components emerged for both groups, again suggesting that relative weighting of the circumstances that formed each domain were common to women and men. Men’s greater focus on self-confidence does harken back to the competitiveness of traditional masculinity. Less stereotypically, contextual determinants (relationship with family and belonging to a community) were ranked equally by both groups, and of lower importance than personal characteristics such as illness and function. Findings also diverge from theoretical assumptions about the centrality of social relations to women in particular [28]. We can only speculate as to reasons for this divergence. Traditional gender norms, such as the importance of social connectedness to women’s well-being and of function and physical health to men, that have been identified in the past [28], may be diminishing. Perhaps this signals evolving constructs of health and a decrease in gender divergent thinking. Such fluctuation over time does align with what seems true intuitively and is in keeping with conceptualizations of gender as being dynamic. Over time (and place), meanings attributed to measures will evolve as social circumstances change, and will develop differently for different groups.

This study is not without limitations. It used a ‘sample of convenience’. Those choosing to participate in this online survey may well have been healthier or more positive in their outlook on life. Despite a large number of responses overall, their age and sex distributions were not equal across categories. We have reported briefly on variability of findings with age to demonstrate that group characteristics other than gender are at play when adults make subjective determinations about health. We do not, however, want to over-analyze age-based data for several reasons. First, we have arbitrarily divided the cohort into three groups, age 25–44, 45–64 and 65 + years, however proportions of respondents are not equal for each 10 year age subcategory (e.g. there were more participants age 25–34 than 35–44). Second, our primary aim was to examine gender differences in meanings of measures. Our findings do suggest that further studies powered to examine age intersecting with sex might deepen understanding of group-level meanings of subjective health measures. Similarly, geographic location may well be a proxy for other commonalities that shape subjective ratings of health and that we could not assess but that merit future consideration. As with any cross-sectional research we cannot generalize or assume causality. Of particular importance, we have been guided by past research in selecting which characteristics men and women might weigh when assessing their subjective health. Earlier in this paper we suggested that what could not be determined using survey methods was whether there are other important precursors of subjective health ratings (e.g. aspects of power and control at home and work, or early adversity) that would differentiate men’s and women’s framing of subjective health. We recommend a qualitative exploration of potential characteristics underlying SRH and SRMH and of intersecting marginalizations of sex and socioeconomic status, race, location, power, age, etc.

After making SRH assessments participants were offered a list of circumstances and asked what they had considered in rating their health. Men and women included most of the listed factors. We cannot determine whether all these factors were truly weighed prior to subjectively rating health. Put another way, might switching the order of questioning have engendered deeper reflection among all? On the other hand, the list of possible circumstances underlying SRH did appear before participants rated their mental health so for that rating triggering of areas to consider would have been experienced by all. Nevertheless, large gender differences in SRMH emerged and, of particular note, connection with family and friends and behaviors was significantly more important in shaping women’s SRMH.

What this study does reinforce is the need for and value of health indicators with unambiguous and common meanings. Our findings show that what SRH and SRMH measure is rsomewhat, but not completely consistent across sex/gender. Divergence of current results from earlier ones is in keeping with gender being dynamic and fluctuating with changes in social norms and realities over time. In general, the existence of group-level differences in interpretation of survey questions may jeopardize reliability of analyses of that data. Because SRH and, to a lesser extent, SRMH are among the most ubiquitous of health survey data collected it is of particular importance that these indicators measure the same thing for all participants. To uncover group-level variability in how participants interpret measures, surveys might add questions such as: how do you rate your health now; what shaped your rating (e.g. comparisons with others of your age, your expectations re longevity, your past health, etc.). Secondary clarifying questions could be added asking participants about their interpretation of the meanings of measures. For example, asking about SRH could be followed by asking what the respondent considered when making their rating, using a list of items such as those we have found to be of importance like mental health, life threatening illnesses, function, or social connections. Although survey developers may balk at lengthening questionnaires, exchanging brevity for accuracy is required if responses to key questions will otherwise be questionable.

Finally, our perhaps naïve hope was to explain the paradox of women’s poorer SRH alongside greater longevity. Solving that enigma remains for others although we can say that sex/gender differences in SRH and SRMH are real and not artefactual.

## Data Availability

Data can be made available upon reasonable request to the corresponding author and after ethics amendments to allow such access.

## Abbreviations

SRH: Self-rated health
SRMH: Self-rated mental health
